# Green Tea Ameliorates Hyperglycemia by Promoting the Translocation of Glucose Transporter 4 in the Skeletal Muscle of Diabetic Rodents

**DOI:** 10.3390/ijms20102436

**Published:** 2019-05-16

**Authors:** Manabu Ueda-Wakagi, Hironobu Nagayasu, Yoko Yamashita, Hitoshi Ashida

**Affiliations:** 1 Department of Agrobioscience, Graduate School of Agricultural Science, Kobe University, Nada-ku, Kobe, Hyogo 657-8501, Japan; mana5998bu@affrc.go.jp (M.U.-W.); Nagayasu_Hironobu@lotte.co.jp (H.N.); yoko.y@crystal.kobe-u.ac.jp (Y.Y.); 2 National Agriculture and Food Research Organization, National Food Research Institute, Tsukuba, Ibaraki 305-8642, Japan

**Keywords:** green tea, diabetes, skeletal muscle, glucose transporter 4

## Abstract

It is known that green tea helps prevent obesity and diabetes mellitus. In this study, we aimed to determine whether green tea ameliorates hyperglycemia and the mechanism involved in diabetic rodents. Green tea consumption reduced blood glucose and ameliorated glucose intolerance, which was assessed using an oral glucose tolerance test in both streptozotocin-induced type 1 diabetic rats and type 2 diabetic KK-A^y^ mice. Green tea also reduced the plasma fructosamine and glycated hemoglobin concentrations in both models. Furthermore, it increased glucose uptake into the skeletal muscle of both model animals, which was accompanied by greater translocation of glucose transporter 4 (GLUT4). Moreover, epigallocatechin gallate (EGCG), the principal catechin in green tea, also ameliorated glucose intolerance in high-fat diet-induced obese and diabetic mice. These results suggest that green tea can ameliorate hyperglycemia in diabetic rodents by stimulating GLUT4-mediated glucose uptake in skeletal muscle, and that EGCG is one of the effective compounds that mediate this effect.

## 1. Introduction

Type I diabetes mellitus (T1DM) is a severe disease characterized by a loss of insulin secretion from pancreatic β-cells [1]. Insulin is the most important hormone for glucose homeostasis, and it regulates the uptake and metabolism of glucose and other nutrients [2]. For example, insulin stimulates glucose uptake into skeletal muscle and adipose tissue, facilitates glycogen synthesis, and suppresses gluconeogenesis. It also stimulates lipogenesis and inhibits the hepatic hydrolysis of triglycerides to liberate glycerol and fatty acids. Therefore, a reduction in insulin secretion causes body weight loss, hyperglycemia, and dyslipidemia. Hyperglycemia is the hallmark of diabetes mellitus (DM), and chronic oversupply of glucose causes protein glycation and oxidative stress, which plays a major role in the development of diabetic complications [3]. Thus, blood glucose control using exogenous insulin or insulin-mimetic agents is very important for T1DM patients.

In contrast, type 2 diabetes mellitus (T2DM) is characterized by insulin resistance in target tissues, including the liver, skeletal muscle, and adipose tissue. Insulin resistance is frequently associated with obesity and also results in hyperglycemia [2]. Hypertrophied adipocytes secrete a number of adipocytokines, including leptin, resistin, and tumor necrosis factor-α (TNF-α), which have metabolic effects [4,5,6]. Many studies have also shown that adipocytokines induce insulin resistance, not only in adipocytes but also in skeletal muscle and the liver, causing reductions in glucose uptake and metabolism [6]. Therefore, the amelioration of insulin resistance is an important target for therapy in T2DM patients.

Insulin administration ameliorates hyperglycemia and lipid abnormalities in many DM patients, but it is difficult and inconvenient for the patients to inject insulin themselves. Consequently, many studies have been carried out to determine whether DM could be treated or prevented using special diets or beverages. Green tea is a candidate for being an antidiabetic beverage because it is well known that it and some of its components, catechins, prevent hyperglycemia [7,8,9]. However, their efficacy at ameliorating hyperglycemia and the underlying mechanism of this effect have not been fully characterized.

Catechins from tea suppress the absorption of glucose from the small intestine by inhibiting α-glucosidase activity [10]. However, our previous study showed that drinking green or black tea for 14 weeks prevented hyperglycemia and obesity without the inhibition of α-glucosidase activity [11]. In the same study, we showed that green or black tea suppressed high-fat diet (HFD)-induced hyperglycemia and insulin resistance by maintaining GLUT4 expression and stimulating glucose uptake, which involved the translocation of GLUT4 to the plasma membrane of muscle. In addition, in rats fed normal chow, consumption of green tea for three weeks was associated with greater glucose uptake and the translocation of GLUT4 in skeletal muscle, but not in adipose tissue [12].

GLUT4 is specifically expressed in skeletal muscle and adipose tissue and plays a pivotal role in the maintenance of blood glucose levels in the postprandial state [13]. When the blood glucose concentration rises, insulin is secreted from pancreatic β-cells, binds to the insulin receptor, and activates the insulin signaling pathway, which promotes the translocation of GLUT4 from intracellular vesicles to the plasma membrane [13]. Recently, we determined the molecular mechanism involved in the induction of GLUT4 translocation by epigallocatechin gallate (EGCG), the principal catechin in green tea [14,15], finding that it promoted GLUT4 translocation through both the phosphoinositol 3-kinase (PI3K) and AMP-activated protein kinase (AMPK) pathways. We also showed that oral administration of EGCG ameliorated postprandial hyperglycemia accompanied by GLUT4 translocation through the same mechanisms in ICR mice. These results imply that green tea could ameliorate the hyperglycemia of T1DM and T2DM by stimulating glucose uptake accompanied by GLUT4 translocation in the skeletal muscle of diabetic rodents.

In the present study, we aimed to determine the effects of green tea consumption on hyperglycemia and glucose uptake in streptozotocin (STZ)-induced T1DM rats and KK-A^y^ mice, a model of T2DM. We also aimed to determine whether oral administration of EGCG would ameliorate the glucose intolerance of HFD-induced obese and diabetic C57BL/6 mice.

## 2. Results

### 2.1. Green Tea Ameliorated Hyperglycemia and Glucose Intolerance in T1DM Rats

**Figure 1:**
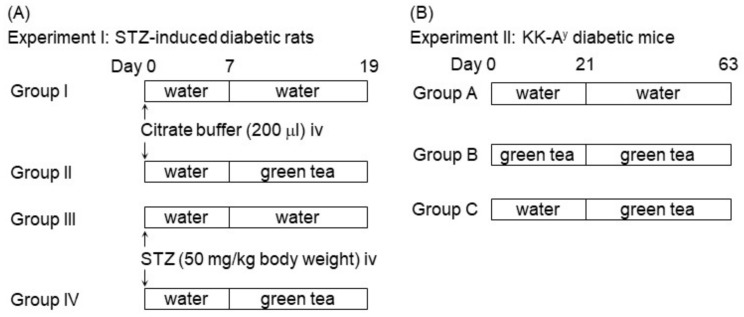
Experimental schedule. (**A**) Experiment I: Male Wistar/ST rats (6 weeks old) were allocated to two groups (*n* = 10 each), one of which was injected with streptozotocin (STZ) (Groups III and IV) and the other with citrate buffer only (Groups I and II) for 7 days. Each group was further divided into two subgroups (*n* = 5 each), one of which was administered green tea (Groups II and IV), while the other drank only water (Groups I and III). During the feeding period, body weight and blood glucose concentration were measured on Days 0, 7, 10, and 13. Then the rats were killed on Day 19. (**B**) Experiment II: KK-A^y^ mice (4 weeks old) were allocated to three groups (*n* = 7 each) on Day 0. Group A received water from the first day on, Group B received green tea from the first day on, and Group C received green tea from Day 21 on, after the appearance of hyperglycemia. During the feeding period, body weight and blood glucose concentration were measured weekly, and the mice were sacrificed on Day 63.

We first investigated the effects of green tea on the hyperglycemia and glucose intolerance of STZ-induced T1DM rats (Figure 1A). The body weights of the STZ-injected rats were significantly lower than those of the control rats (Group III (STZ + water), 171 ± 10 g; Group IV (STZ + green tea), 169 ± 6 g; Group I (nondiabetic water), 279 ± 7 g; Group II (nondiabetic green tea), 283 ± 3 g) at the end of the experiment. However, supplementation of green tea did not alter the body weights. In addition, food intake was not affected by STZ injection, although water intake was higher in the STZ-injected groups than in the control groups (data not shown). These results indicate that green tea did not ameliorate STZ-induced body weight loss.

**Figure 2:**
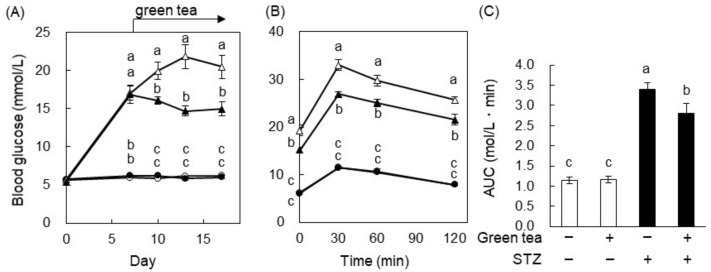
Green tea ameliorated hyperglycemia and glucose intolerance in STZ-induced type I diabetes mellitus (T1DM) rats. The rats were treated as described in Figure 1A. (**A**) Their blood glucose concentrations were measured 0, 7, 10, 13, and 17 days after the STZ injection. (**B**) An oral glucose tolerance test (OGTT) was performed on Day 17. The rats were orally administered glucose (2 g/kg body weight) after 18 h of fasting, and their blood glucose concentrations were measured 0, 30, 60, and 120 min later. Here, ○: water; ●: green tea; △: STZ + water; ▲: STZ + green tea. (**C**) The area under the curve (AUC) of the OGTT was calculated. Data are expressed as the means ± standard error (SE) (*n* = 5). Values sharing the same letters were not significantly different (Tukey–Kramer multiple comparison test; *p* < 0.05).

To investigate the effect of green tea on hyperglycemia, we measured the fasting blood glucose level during the feeding period. The injection of STZ increased the blood glucose concentration, as expected. On Day 7, this reached about 16.6 mmol/L in the STZ-injected groups, whereas it was ~5.56 mmol/L in the control groups (Figure 2A). Green tea was then administered from Day 7 on, and was found to significantly ameliorate STZ-induced hyperglycemia but not to cause hypoglycemia in the control group. On Day 17, an oral glucose tolerance test (OGTT) was performed. The blood glucose of the STZ + green tea group was significantly lower than that of the STZ + water group at all time points, including 0 min (Figure 2B). Since the blood glucose response profile was similar between the STZ-injected + water and STZ + green tea groups, we calculated the area under the curve (AUC) using the data in Figure 2B and found that the AUC of the STZ + green tea group was significantly lower than that of the STZ + water group (Figure 2C). In the nondiabetic rats, green tea did not affect glucose tolerance. The plasma insulin level was measured 30 min after glucose administration, because STZ is known to cause pancreatic failure with insulin secretion. The plasma insulin levels of the STZ-injected groups were significantly lower than those of the control groups (Group III (STZ + water), 0.194 ± 0.021 μg/L; Group IV (STZ + green tea), 0.197 ± 0.021 µg/L; Group I (nondiabetic water), 0.324 ± 0.025 µg/L; Group II (nondiabetic green tea), 0.316 ± 0.023 µg/L). However, green tea did not ameliorate the STZ-induced reduction in the plasma insulin level 30 min after glucose administration. These results suggest that green tea ameliorated STZ-induced hyperglycemia and glucose intolerance without affecting insulin secretion from pancreatic β-cells.

Because diabetic patients also demonstrate abnormalities in lipid metabolism that are associated with certain complications, we also measured plasma non-esterified fatty acids (NEFAs), total cholesterol (TC), and triacylglycerol (TG) concentrations in the rats. In particular, NEFAs have been reported to induce insulin resistance in the skeletal muscle and liver [16]. As shown in Table 1, the plasma NEFAs and TG concentrations were significantly higher in the STZ + water group than in the nondiabetic water group. However, green tea ameliorated STZ-induced increases in both plasma NEFAs and TG. Neither STZ nor green tea affected plasma TC concentrations.

**Figure 3:**
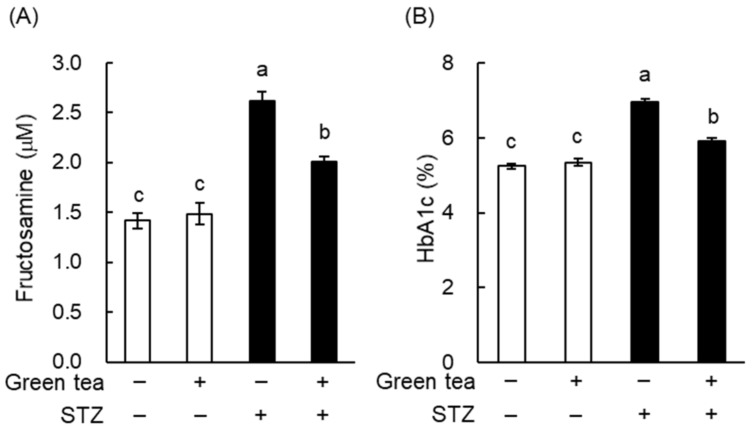
Green tea consumption reduced plasma fructosamine and glycated hemoglobin (HbA1c) concentrations in STZ-induced T1DM rats. Rats were treated as described in Figure 1A. (**A**) Plasma fructosamine was measured using the nitro blue tetrazolium (NBT) colorimetric method, as described in “Materials and Methods”. (**B**) Plasma HbA1c was measured using a commercial test kit. Data are expressed as the means ± SE (*n* = 5). Values sharing the same letters were not significantly different (Tukey-Kramer multiple comparison test; *p* < 0.05).

Fructosamine and glycated hemoglobin (HbA1c) concentrations reflect the degree of protein glycosylation and are widely used in the diagnosis and monitoring of diabetes [17]. As shown in Figure 3, plasma fructosamine and HbA1c were significantly higher in the STZ + water group than in the nondiabetic mice. However, green tea significantly ameliorated STZ-induced increases in plasma fructosamine and HbA1c. These results suggest that green tea may be able to prevent or delay the progression of diabetic complications in T1DM patients.

### 2.2. The Mechanism of the Anti-Hyperglycemic Effect of Green Tea in T1DM Rats

**Figure 4:**
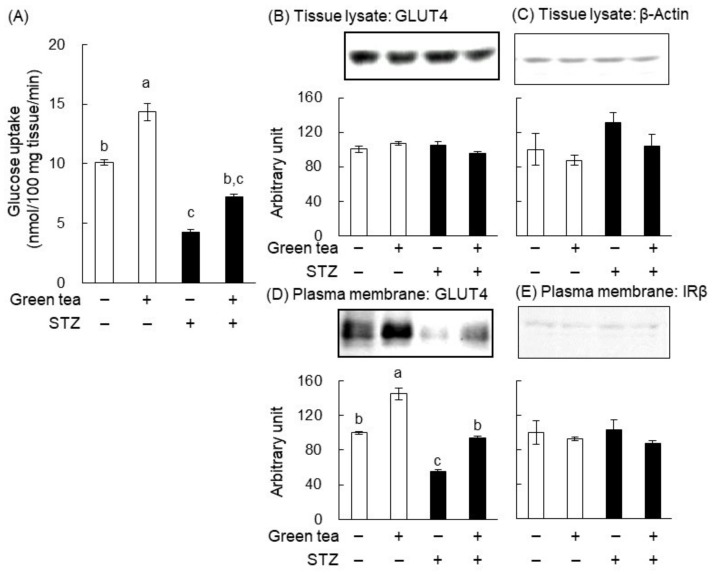
Green tea consumption increased glucose uptake and glucose transporter 4 (GLUT4) translocation in the skeletal muscle of STZ-induced T1DM rats. Rats were treated as described in Figure 1A. At the end of the treatment, the rats were administered glucose (2 g/kg body weight) and were sacrificed 1 h later. Skeletal muscle was cut into small pieces of 50 mg each, and 3-*O*-methyl-D-[1-^3^H] glucose (3-OMG) uptake was measured (**A**) as described in “Materials and Methods”. The expressions of GLUT4 and β-actin in tissue lysates (**B**,**C**) and the quantities of GLUT4 and the β-subunit of the insulin receptor (IRβ) present in the plasma membrane fractions (**D**,**E**) were determined by western blotting analysis, as described in “Materials and Methods”. In (**B***–***E**), the upper panels show typical immunoblots and the lower panels show the density of each band for all rats (*n* = 5). Data are expressed as the means ± SE. Values sharing the same letters were not significantly different (Tukey–Kramer multiple comparison test; *p* < 0.05).

In our previous studies, we showed that green tea increased glucose uptake and GLUT4 translocation in skeletal muscle [11,15]. Therefore, we determined the effect of green tea on glucose uptake into the skeletal muscle of STZ-induced diabetic rats (Figure 4A). Glucose uptake was significantly lower in the STZ + water group than in the nondiabetic water group, but this lower glucose uptake tended to be ameliorated by the consumption of green tea. In addition, green tea significantly increased glucose uptake in nondiabetic rats.

We next investigated the effect of green tea consumption on GLUT4 translocation in the skeletal muscle of STZ-induced T1DM rats. The protein expression levels of GLUT4 and β-actin (as a loading control) in tissue lysates were similar in all of the groups using western blot analysis (Figure 4B,C). However, the quantity of GLUT4 in the plasma membrane fraction of the muscle was significantly lower in the STZ + water group than in the nondiabetic water group (Figure 4D). Furthermore, green tea consumption was associated with a near-normalization of this defect (Figure 4D). In addition, green tea significantly increased the translocation of GLUT4 in the nondiabetic rats (Figure 4D). On the other hand, the level of the β-subunit of the insulin receptor (IRβ) (as the loading control for the plasma membrane fraction) was not altered (Figure 4E). These results imply that green tea ameliorated hyperglycemia in T1DM rats by promoting GLUT4 translocation in skeletal muscle, thereby increasing glucose disposal into this tissue.

### 2.3. Green Tea Ameliorated Hyperglycemia and Glucose Intolerance in T2DM Mice

We next investigated the effect of green tea on the hyperglycemia of KK-A^y^ mice (Figure 1B). Because KK-A^y^ mice develop genetically determined obesity, in addition to diabetes [18], their body weight was monitored during the feeding period. There were no differences in body weight among the three treatment groups (data not shown), suggesting that green tea consumption did not affect growth or adiposity in KK-A^y^ mice. At the end of the treatment period, epididymal adipose tissue weight was slightly reduced by green tea without significance (Group A, 1.30 ± 0.23 g; Group B, 1.07 ± 0.12 g; and Group C, 1.00 ± 0.11 g). There was no change in other white adipose tissue weights. These results indicate that green tea did not prevent obesity in KK-A^y^ mice.

**Figure 5:**
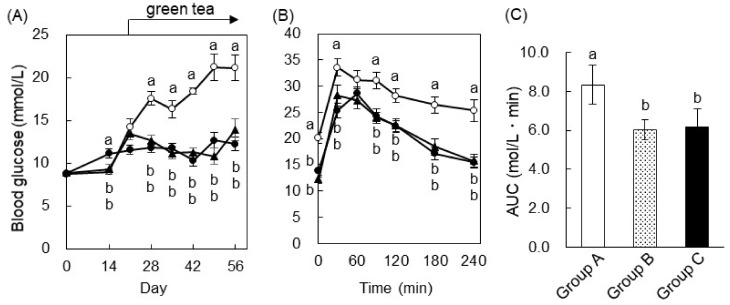
Green tea ameliorated hyperglycemia and glucose intolerance in KK-A^y^ mice, a type 2 diabetes mellitus (T2DM) model. Mice were treated as shown in Figure 1B. (**A**) Blood glucose concentrations were measured weekly during the feeding study. (**B**) An OGTT was performed on Day 56. The mice were orally administered glucose (2 g/kg body weight) 6 h after fasting, and the blood glucose level was measured 0, 30, 60, 120, 180, and 240 min later. Here, ○: Group A; ●: Group B; and ▲: Group C. (**C**) The AUC of the OGTT was calculated. Data are expressed as the means ± SE (*n* = 7). Values sharing the same letters were not significantly different (Tukey–Kramer multiple comparison test; *p* < 0.05).

To investigate the effects of green tea on hyperglycemia, the fasting blood glucose level was measured during the feeding period (Figure 5A). In Group A (drinking water), blood glucose increased continuously, whereas in Group B (drinking green tea from Day 0), the increase in blood glucose was less, only reaching ~11.11 mmol/L. In Group C (drinking green tea from Day 21), blood glucose increased from Day 0 (8.87 ± 0.21 mmol/L) to Day 21 (13.44 ± 0.79 mmol/L), but once the mice started drinking the green tea, their blood glucose dropped to ~11.11 mmol/L and there was no further increase. These results indicate that green tea possessed not only preventive but also therapeutic effects on hyperglycemia in KK-A^y^ mice.

On Day 56, an OGTT was performed. In Group A, blood glucose reached a maximum 30 min after glucose administration and thereafter gradually decreased until 180 min, although hyperglycemia remained (Figure 5B). In the green tea-drinking groups, blood glucose increased after glucose loading to the same extent as in Group A. However, green tea drinkers demonstrated a reduction in blood glucose from its maximum concentration to basal by 180 min. It is noteworthy that the highest blood glucose concentration in Group B was observed 60 min after glucose loading. As shown in Figure 5C, the AUCs of Groups B and C were significantly lower than those of Group A. These results suggest that green tea consumption was associated with a rapid decrease in blood glucose after an oral glucose challenge but had no effect on the initial acute increase in blood glucose.

**Figure 6:**
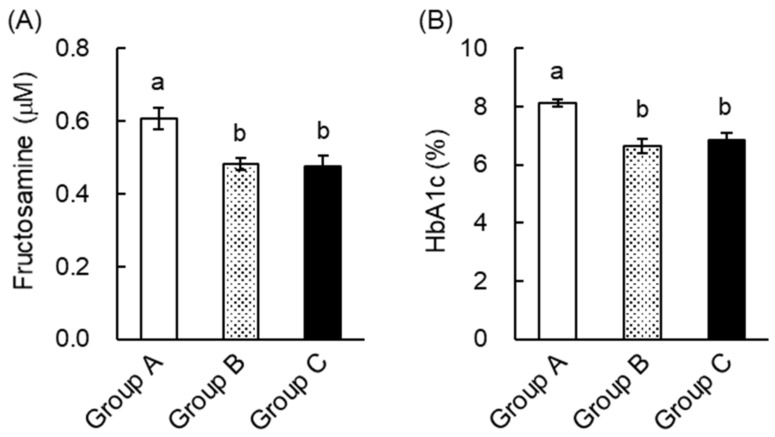
Green tea consumption reduced plasma fructosamine and HbA1c concentrations in KK-A^y^ mice. Mice were treated as shown in Figure 1B. (**A**) Plasma fructosamine was measured using the NBT colorimetric method, as described in “Materials and Methods”. (**B**) Plasma HbA1c was measured using a commercial test kit. Data are expressed as the means ± SE (*n* = 7). Values sharing the same letters were not significantly different (Tukey–Kramer multiple comparison test; *p* < 0.05).

When we measured plasma lipid concentrations, we found that plasma TG in Groups B and C was significantly lower than in Group A (Table 2). However, green tea consumption did not alter plasma TC or NEFA levels. These results indicate that green tea ameliorated hypertriglyceridemia in KK-A^y^ mice. TNF-α is known to be involved in the induction of insulin resistance. Measurement of plasma TNF-α by ELISA showed that green tea drinkers tended to have lower concentrations (data not shown). This finding suggests that a reduction in TNF-α may also have contributed to the amelioration of hyperglycemia through green tea consumption. To estimate the extent of protein glycation, we also measured plasma fructosamine and HbA1c concentrations. As shown in Figure 6, these were significantly lower in Groups B and C than in Group A. These results suggest that green tea may be capable of both preventing and ameliorating diabetic complications in T2DM patients.

### 2.4. The Mechanism of the Anti-Hyperglycemic Effect of Green Tea in T2DM Mice

**Figure 7:**
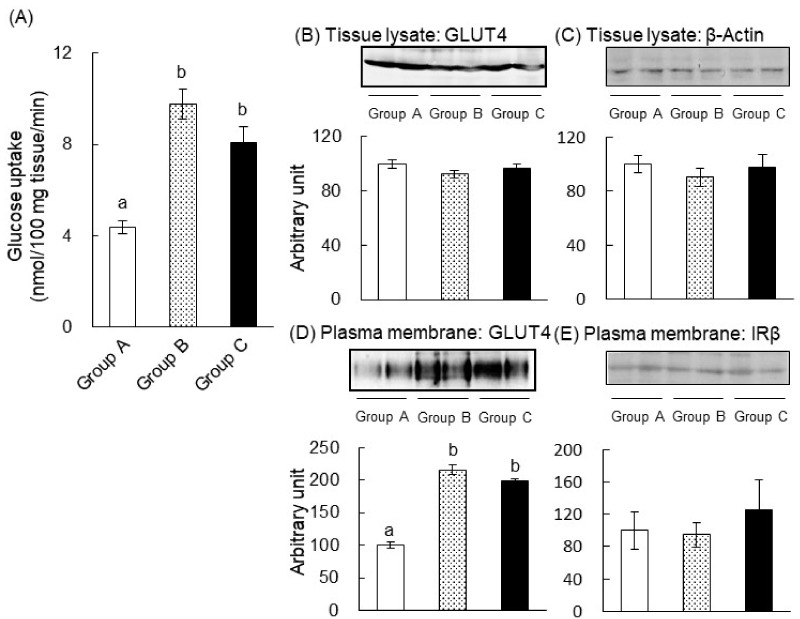
Green tea consumption increased glucose uptake and GLUT4 translocation in the skeletal muscle of KK-A^y^ mice. Mice were treated as shown in Figure 1B. On Day 63, the mice were orally administered glucose (2 g/kg body weight) and were sacrificed 1 h later. (**A**) Skeletal muscle was cut into small pieces of 50 mg each, and 3-OMG uptake was measured as described in “Materials and Methods”. The expressions of GLUT4 and β-actin in tissue lysates (**B**,**C**) and the quantities of GLUT4 and IRβ present in the plasma membrane fractions (**D**,**E**) were determined through western blot analysis, as described in “Materials and Methods”. In (**B**–**E**), the upper panels show typical immunoblots and the lower panels summarize the density of each band in all mice (*n* = 7). Data are expressed as the means ± SE. Values sharing the same letters were not significantly different (Tukey–Kramer multiple comparison test; *p* < 0.05).

To understand the molecular mechanism through which green tea ameliorates hyperglycemia, we measured glucose uptake into the skeletal muscle 60 min after glucose administration (Figure 7A). Green tea significantly increased glucose uptake into the muscle of Groups B (2.2-fold) and C (1.9-fold) compared to Group A. This indicates that green tea could not only prevent but also ameliorate insulin resistance in T2DM mice by promoting glucose uptake into the skeletal muscle. We also assessed GLUT4 translocation from intracellular vesicles to the plasma membrane. The amount of GLUT4 and β-actin (as a loading control) in tissue lysates was similar in each group (Figure 7B,C). However, green tea consumption significantly increased the amount of GLUT4 present in the plasma membrane fraction, indicating an upregulation of translocation, in Groups B (2.2-fold) and C (2.0-fold) compared to Group A (Figure 7D), though the level of IRβ, as the loading control for the plasma membrane fraction, remained unchanged. Therefore, the anti-hyperglycemic actions of green tea were likely to be, at least in part, due to an increase in GLUT4-mediated glucose uptake.

### 2.5. EGCG Ameliorated Glucose Intolerance in High-Fat Diet-Induced Obese, Diabetic Mice

**Figure 8:**
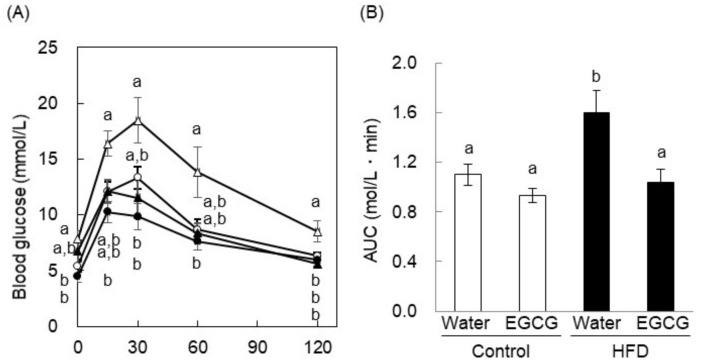
EGCG ameliorated glucose intolerance in high-fat diet-induced obese and diabetic mice. (**A**) After 13 weeks of high-fat diet (HFD) feeding, mice were orally administered glucose (2 g/kg body weight) after 18 h of fasting, and their blood glucose concentrations were measured 0, 30, 60, and 120 min later. Here, ○ and ●: control diet-fed mice; △ and ▲: HFD-fed mice; ● and ▲: EGCG (75 mg/kg body weight was orally administered 60 min before glucose administration); ○ and △: the same volume of water was administered. (**B**) The AUC of the OGTT was calculated. Data are expressed as the means ± SE (*n* = 4). Values sharing the same letters were not significantly different (Tukey–Kramer multiple comparison test; *p* < 0.05).

We also investigated the effects of EGCG, which is the most abundant catechin in green tea, in HFD-induced obese, diabetic C57BL/6 mice. As expected, the blood glucose concentration of the HFD + water group was significantly higher than that of the control diet + water group (Figure 8). In both groups that received water only, blood glucose increased during the first 15 min after glucose administration, peaked after 30 min, and thereafter decreased. In the green tea-drinking groups, blood glucose increased during the first 15 min and thereafter decreased. In the HFD-fed groups, EGCG significantly reduced the blood glucose concentration at the 15-min time point versus the control group, and EGCG also significantly lowered the AUC value compared to the HFD-fed control group. This result suggests that EGCG ameliorated glucose intolerance in the HFD-induced obese, diabetic mice.

## 3. Discussion

Green tea is a popular traditional beverage in Japan and China, but it has also become popular around the world, because it has been shown to have various health benefits and may prevent a number of diseases. Epidemiologic studies have shown that the consumption of green tea may reduce body weight and fat accumulation by improving glucose and lipid metabolism [19], and it has also been shown that green tea extract and EGCG ameliorate hyperglycemia and insulin resistance in humans and animals [7,8,9,11,12,19,20]. However, the nature and mechanisms of the beneficial effects of green tea and tea catechins on hyperglycemia are not fully understood. In this study, we demonstrated that green tea ameliorated hyperglycemia in STZ-induced T1DM rats, KK-A^y^ mice (a model of T2DM), and HFD-fed C57BL/6 obese and diabetic mice (Figure 2, Figure 5 and Figure 8). In T1DM rats and T2DM mice, green tea also reduced high plasma fructosamine and HbA1c concentrations (Figure 3 and Figure 6), suggesting that green tea may be capable of ameliorating the diabetic complications associated with chronic hyperglycemia.

We also identified a possible mechanism for the anti-hyperglycemic effect of green tea and its major polyphenol component, EGCG, because they increased the translocation of GLUT4 to the plasma membrane of muscle, implying greater glucose uptake into this tissue (Figure 4 and Figure 7). This is the first report showing that an increase in glucose uptake by skeletal muscle may be involved in the amelioration of hyperglycemia through green tea in animal models of diabetes. Our findings are especially significant because they indicate that green tea ameliorated hyperglycemia in models of both principal types of diabetes. The present results are also consistent with those of a previous study that showed that green tea catechin ameliorated hyperglycemia by improving adipose insulin sensitivity in KK-A^y^ mice [21].

The stimulation of glucose uptake into muscle is the most important means whereby insulin reduces postprandial hyperglycemia, and GLUT4 plays a pivotal role in maintaining the blood glucose level [22]. In the present study, we found evidence that green tea consumption increased glucose uptake by increasing GLUT4 translocation in hyperglycemic animals. This mechanism was consistent with our previous findings that green or black tea prevented hyperglycemia by stimulating glucose uptake and GLUT4 translocation in the skeletal muscle of HFD-fed mice [11]. In addition, it has been shown that drinking oolong or pu-eat tea for seven days promotes GLUT4 translocation [23]. These results suggest that not only EGCG, but also other compounds in tea, may promote GLUT4 translocation. Indeed, previous studies [21,24,25,26] have demonstrated that certain food components, in particular polyphenols, promote glucose uptake by peripheral tissues. Moreover, we found a single oral administration of EGCG decreased postprandial hyperglycemia in HFD-fed mice (Figure 8). This result suggests that EGCG had the potency to suppress blood glucose spikes in diet-induced obese and diabetic conditions. 

The green tea catechin EGCG promotes glucose uptake and GLUT4 translocation in L6 myotubes at a concentration of 1 nM [15] through activation of the PI3K and AMPK pathways [14]. EGCG has also been shown to activate these kinases in 3T3-L1 adipocytes, but this requires a concentration of 50 μM [27]. It is known that the bioavailability of EGCG is low: For example, after the oral administration of 100 mg of EGCG to SD rats, the *C_max_* of EGCG was 11.0 μg/L (about 24 nM) [28]. In our previous study, 0.97 nM of EGCG aglycone was detected in the plasma after green tea consumption for seven days [29]. This indicated that EGCG promoted GLUT4 translocation and glucose uptake in skeletal muscle at achievable concentrations. In the present study, we did not measure catechin concentrations in the plasma and muscle, but these measurements should be performed in future studies. However, because the disposal of blood glucose into peripheral tissues, especially skeletal muscle, is critical for the amelioration of hyperglycemia in diabetes mellitus [1], our findings represent a significant step forward in the characterization of the antidiabetic effects of green tea.

In T2DM, the development of insulin resistance precedes hyperglycemia and diabetic complications. Insulin resistance in skeletal muscle involves the inactivation of Akt and/or PI3K [2], and our previous study showed that the consumption of an HFD for 14 weeks reduced the expression of GLUT4, IRβ, and AMPK, but also reduced the translocation of GLUT4 in the plasma membrane fraction of skeletal muscle [11]. Green and black tea had the potential to prevent the reduction of GLUT4, IRβ, and AMPK expression and to maintain GLUT4 translocation to the plasma membrane, which were considerable anti-hyperglycemic effects [11]. Moreover, we also demonstrated that EGCG promoted GLUT4 translocation into both insulin-resistant L6 myotubes and myotubes deficient in the insulin receptor [15]. In the present study, we showed that EGCG ameliorated glucose intolerance in HFD-induced obese and diabetic mice (Figure 8). Taken together, these results suggest that green tea and EGCG might ameliorate insulin resistance and promote GLUT4 translocation and glucose uptake into the skeletal muscle of diabetic animals via the PI3K and AMPK pathways without phosphorylation of IRβ as the upstream event of GLUT4 translocation. This is an important issue to clarify in the future. Further study is needed to confirm the exact molecular mechanism by which green tea and its catechins promoted GLUT4 translocation in the diabetic model animals in the same manner as in the normal-died fed animals. 

A reduction in hyperglycemia is likely to help prevent the development of diabetic complications. Chronic hyperglycemia induces the glycation of proteins in the plasma and tissues, and this is associated with the development of oxidative stress, which mediates many complications [3]. For example, the oxidation of low-density lipoprotein (LDL) is a key component of atherogenesis [30]. In the present study, green tea consumption significantly reduced plasma fructosamine and HbA1c in both T1DM rats (Figure 3) and T2DM mice (Figure 6). Hypertriglyceridemia is associated with changes in the composition of LDL, and TG-rich LDL facilitates atherogenesis similarly to oxidized LDL [31]. We showed that green tea significantly reduced plasma TG in T2DM mice (Table 2). In addition, it is well documented that green tea and catechins ameliorate oxidative stress and lipid abnormalities [32,33]. Taken together, these results imply that green tea has the potential to ameliorate or prevent complications, because by ameliorating hyperglycemia, it also reduces protein glycation and oxidative stress. Thus, green tea is likely to prevent and ameliorate the complications of diabetes mellitus through various mechanisms.

## 4. Materials and Methods

### 4.1. Chemicals and Reagents

A green tea extract, “Theafrane-30A^TM^”, was obtained from Itoen Inc. (Tokyo, Japan). EGCG (98% purity) was kindly provided by Dr. Suong-Hyu Hyon (Kyoto University, Japan) and stored at −20 °C. Glucose, TG, NEFAs, and TC test kits were purchased from Wako Pure Chemical Industries, Ltd. (Osaka, Japan). An HbA1c test kit was obtained from Sigma-Aldrich Co. (St. Louis, MO, USA). For western blot analysis, polyclonal anti-GLUT4 and anti-IRβ (Santa Cruz Biotechnology, Inc., Santa Cruz, CA, USA), anti-β-actin and anti-rabbit IgG (Sigma-Aldrich Co., St. Lois, MO, USA), and anti-goat IgG (Wako Pure Chemical Industries, Ltd.) antibodies were used. A polyvinylidene difluoride (PVDF) membrane was purchased from Amersham Biosciences Ltd. (Buckinghamshire, UK). An ImmunoStar^®^ LD western blotting detection reagent was from Wako Pure Chemical Industries, Ltd. All other reagents were of the highest grade available.

### 4.2. Animal Treatments

The animal procedures were approved by the Kobe University Institutional Animal Care and Use Committee and were carried out according to the guidelines for animal experimentation of the Kobe University Animal Experimentation Regulator (permission #13-4-17 and #13-4-18 (from 6 March 2001) and #21-07-02 (from 9 April 2010)). Male Wistar/ST rats (6 weeks old, purchased from Japan SLC, Inc., Shizuoka, Japan) were maintained at 23 ± 2 °C on a 12-h light/dark cycle (lights on at 09:00). The rats were acclimated for 7 days and had free access to tap water and a commercial chow diet. As shown in Figure 1A, the rats were divided into four groups of five each: Group I, nondiabetic, administered water; Group II, nondiabetic rats, administered green tea; Group III, STZ-induced T1DM rats, administered water; and Group IV, STZ-induced T1DM rats, administered green tea. To induce T1DM, STZ was freshly prepared in 0.05 M of citrate buffer, pH 4.5, and was intravenously injected into the rats at doses of 50 mg/kg body weight. Nondiabetic rats were administered an equal volume of citrate buffer alone. Theafrane-30A^TM^ solution, which was dissolved in water at 2 g/L, was given to the appropriate groups instead of water from 7 days after the injection of STZ. The composition of bioactive compounds in the Theafrane-30A^TM^ was 0.3% (+)-catechin, 1.7% (−)-epicatechin, 1.3% (−)-gallocatechin, 8.3% (−)-epigallocatechin, 0.3% (−)-catechin gallate, 2.8% (−)-epicatechin gallate, 0.5% (−)-gallocatechin gallate, 16.5% (−)-EGCG, and 6.3% caffeine.

Body weight, food intake, and water intake were measured every 3 days. To measure blood glucose concentrations during the feeding period, blood was collected from a tail vein of rats into tubes containing sodium heparin as an anticoagulant on Days 0, 7, 10, and 13, after 6 h of fasting. On Day 17, an OGTT was performed as follows: The rats were orally administered glucose solution (2 g/kg body weight) after an 18-h fast, and blood was collected before (0 min), and 30, 60, and 120 min after glucose loading. On Day 19, glucose (2 g/kg body weight) was orally administered to the rats after an 18-h fast, and then the animals were killed by exsanguination following cardiac puncture (under anesthesia using sodium pentobarbital, 60 min after the administration of glucose (2 g/kg body weight)). Blood samples were centrifuged at 1500× *g* for 10 min at 4 °C, and plasma was collected. Muscle was collected from the hindlimbs and was used to assess glucose uptake and GLUT4 translocation.

Male KK-A^y^/Ta Jcl mice (4 weeks old, purchased from CLEA Japan, Inc, Tokyo, Japan) were maintained and acclimated under the conditions described above. As shown in Figure 1B, the mice (4 weeks old) were divided into three groups of seven each: Group A, which received water from the first day; Group B, which received green tea from the first day; and Group C, which received green tea from Day 21. To measure blood glucose concentrations during the feeding period, blood was collected from a tail vein weekly after a 6-h fast. On Day 56, an OGTT was carried out as described above. Blood was collected before (0 min), and 30, 60, 90, 120, 180, and 240 min after glucose loading. On Day 63, the mice were sacrificed 60 min after the oral administration of glucose (2 g/kg body weight). Plasma was obtained as described above. Skeletal muscle was collected from their hindlimbs and was used to assess glucose uptake and GLUT4 translocation.

Male C57BL/6 mice (5 weeks old, 19–21 g; Japan SLC, Shizuoka, Japan) were also maintained and acclimated under the conditions described above. After acclimation, they were randomly allocated to two groups of eight each and were fed an HFD containing 30% lard or an AIN93M-based control diet for 13 weeks. The compositions of the diets used were the same as in our previous study [11]. The mice fasted for 18 h and were then further randomly allocated to two subgroups of four each. They were then orally administered either EGCG (75 mg/kg body weight) or 0.85% NaCl (5 mL/kg body weight) as a vehicle control. After 60 min, an OGTT was performed as described above. Blood was collected from a tail vein before (0 min), and 30, 60, and 120 min after glucose loading. Plasma was then obtained, and glucose concentrations were measured.

### 4.3. Measurement of Plasma Fructosamine, HbA1c, and Lipid Concentrations

The plasma fructosamine concentration was measured using a nitro blue tetrazolium (NBT) colorimetric assay, as described previously [34]. Briefly, plasma samples (50 μL) were added to 1-mL aliquots of carbonate buffer (200 mM sodium carbonate, 2.5 kU/L uricase) containing 0.48 mM NBT and were incubated at 37 °C for 15 min. The fructosamine concentration was calculated using the difference in the absorbance at 530 nm at the 10- and 15-min time points. Plasma HbA1c, TG, NEFA, and TC concentrations were measured using the corresponding commercial test kits, according to the manufacturer’s instructions.

### 4.4. Measurement of Glucose Uptake into Skeletal Muscle

Measurement of the glucose uptake by skeletal muscle was carried out as described previously [11]. Briefly, 1 h after glucose administration, the skeletal muscle was removed from the hindlimbs and cut into small pieces (about 50 mg each). One muscle piece was then pre-incubated in 1 mL of Krebs–Ringer phosphate-HEPES buffer (KRH: 50 mM HEPES, pH 7.4, 137 mM NaCl, 4.8 mM KCl, 1.85 mM CaCl_2_, and 1.3 mM MgSO_4_) for 10 min at 37 °C. Then, 3-OMG (DuPont/NEN Research Products) was added to a final concentration of 6.5 mM (1.85 × 10 ^4^ Bq/assay) and incubated at 37 °C for 2 min. The assay was then terminated by washing it six times with ice cold KRH, and the muscle was solubilized in 1 mL of NCS tissue solubilizer (Amersham Biosciences Ltd., UK). The radioactivity in the muscle was then counted in a liquid scintillation cocktail using a liquid scintillation counter with a scintillation cocktail. To determine the nonspecific uptake, another piece of muscle was pre-incubated with 20 μM of cytochalasin B, a potent inhibitor of glucose transporters.

### 4.5. Assessment of GLUT4 Translocation in Skeletal Muscle

Tissue lysates and a plasma membrane fraction were prepared from the skeletal muscle as described previously [11]. After the determination of protein concentration, aliquots of tissue lysates containing 30 μg of protein and a plasma membrane fraction containing 5 μg of protein were separated by 10% SDS-polyacrylamide gel electrophoresis and transferred to PVDF membranes. The membranes were then blocked using Blocking One^®^ solution (Nacalai Tesque, Kyoto, Japan) for 2 h at room temperature, incubated with primary antibodies overnight at 4 °C, and then incubated with the corresponding horseradish peroxidase-conjugated secondary antibody for 1 h at room temperature. The proteins were visualized using an ImmunoStar^®^ LD and Light-Capture II (ATTO Corp., Tokyo, Japan). The density of the specific band was determined using ImageJ image analysis software (National Institutes of Health, Bethesda, MD, USA).

### 4.6. Statistical Analysis

Data are represented as the means ± standard error (SE). The Tukey–Kramer multiple comparison test was used to determine the significant differences among the experimental groups. Statistical analysis was performed using JMP statistical software version 11.2.0 (SAS Institute, Cary, NC, USA). The level of significance was set as *p* < 0.05.

## 5. Conclusions

In this study, we demonstrated that green tea ameliorated hyperglycemia and reduced protein glycation in both STZ-induced diabetic rats and KK-A^y^ mice. The mechanism of the effect of green tea appeared to involve the stimulation of glucose uptake accompanied by promoting GLUT4 translocation in skeletal muscle. Furthermore, EGCG improved glucose intolerance in HFD-induced obese and diabetic mice. These findings indicate that green tea may be useful in the treatment of diabetes mellitus.

## Figures and Tables

**Table 1:** Green tea ameliorated abnormalities in plasma lipid concentrations in STZ-induced T1DM rats.

	Normal		STZ	
Contents	Water	Green Tea	Water	Green Tea
Triacylglycerol (mg/dL)	27.3 ± 1.61 ^a^	29.9 ± 1.95 ^a^	38.8 ± 2.08 ^b^	27.6 ± 0.97 ^a^
Total cholesterol (mg/dL)	56.2 ± 3.80	54.0 ± 5.05	56.1 ± 4.44	54.1 ± 3.98
NEFAs (meq/dL)	0.38 ± 0.02 ^a^	0.42 ± 0.06 ^a^	1.33 ± 0.05 ^b^	0.88 ± 0.07 ^c^

Rats were treated as shown in Figure 1A, and the concentrations of plasma lipids were measured using the corresponding commercial test kits. Data are expressed as the means ± SE (*n* = 5). Values sharing the same letters were not significantly different (Tukey–Kramer multiple comparison test; *p* < 0.05). NEFAs: non-esterified fatty acids.

**Table 2:** Green tea ameliorated abnormalities in plasma lipids in KK-A^y^ mice.

Contents	Group A	Group B	Group B
Triacylglycerol (mg/dL)	166.4 ± 7.3 ^a^	121.7 ± 5.6 ^b^	118.4 ± 6.3 ^b^
Total cholesterol (mg/dL)	140.8 ± 7.2	133.3 ± 9.3	157.2 ± 7.2
NEFAs (meq/dL)	0.37 ± 0.03	0.31 ± 0.03	0.30 ± 0.02

Mice were treated as shown in Figure 1B, and the concentrations of plasma lipids were measured using the corresponding commercial test kits. Data are expressed as the means ± SE (*n* = 7). Values sharing the same letters were not significantly different (Tukey–Kramer multiple comparison test; *p* < 0.05).

## Abbreviations

AMPK	AMP-activated protein kinase
AUC	Area under the curve
DM	Diabetes mellitus
EGCG	Epigallocatechin gallate
GLUT4	Glucose transporter 4
HbA1c	Glycated hemoglobin
HFD	High-fat diet
IRβ	β-subunit of the insulin receptor
KRH	Krebs–Ringer phosphate-HEPES buffer
LDL	Low-density lipoprotein
NBT	Nitro blue tetrazolium
NEFAs	Non-esterified fatty acids
OGTT	Oral glucose tolerance test
3-OMG	3-*O*-Methyl-D-[1-^3^H] glucose
PI3K	Phosphoinositol 3-kinase
PVDF	Polyvinylidene difluoride
STZ	Streptozotocin
TNF-α	Tumor necrosis factor-α
T1DM	Type I diabetes mellitus
T2DM	Type 2 diabetes mellitus
TC	Total cholesterol
TG	Triacylglycerol
